# Physiological Responses to High‐Intensity Interval Exercise in Hypoxia Among Lean Males and Those With Overweight or Obesity

**DOI:** 10.1002/ejsc.70016

**Published:** 2025-07-18

**Authors:** Zhenhuan Wang, Jia Li, Muhammed M. Atakan, Metodija Kjertakov, Hansen Li, Jujiao Kuang, Michael J. McKenna, Wentao Lin, Yanchun Li, David J. Bishop, Olivier Girard, Xu Yan, Li Peng

**Affiliations:** ^1^ Key Laboratory of General Administration of Sport Southwest University Chongqing China; ^2^ Institute for Health and Sport Victoria University Melbourne Australia; ^3^ Division of Exercise Nutrition and Metabolism Faculty of Sport Sciences Hacettepe University Ankara Türkiye; ^4^ Sarcopenia Research Program Australia Institute for Musculoskeletal Sciences (AIMSS) Melbourne Australia; ^5^ College of Sport Science Zhuhai College of Science and Technology Zhuhai China; ^6^ China Institute of Sport and Health Beijing Sport University Beijing China; ^7^ School of Human Sciences (Exercise and Sport Science) The University of Western Australia Perth Australia; ^8^ Department of Medicine‐Western Health The University of Melbourne Melbourne Australia

**Keywords:** blood glucose, body mass index, cardiorespiratory fitness, exercise, simulated altitude

## Abstract

This study aimed to compare physiological responses to high‐intensity interval exercise (HIIE) in hypoxia and normoxia across different body mass index (BMI) categories. Twenty‐one males, classified as normal‐weight (NW, *n* = 9 and BMI: 22.9 ± 2.3 kg · m^−2^) or overweight/obese (OW, *n* = 12 and BMI: 27.6 ± 2.0 kg · m^−2^), completed graded exercise tests (GXT) in normoxia (FiO_2_ = 20.9%) and hypoxia (FiO_2_ = 14.0%), followed by three randomised HIIE sessions: hypoxia (HY), normoxia matched to hypoxic relative intensity (NR) and normoxia matched to hypoxic absolute intensity (NA). Blood samples were collected at baseline, immediately post‐HIIE and at 3 and 24 h post‐exercise. Both NW and OW groups had significant reductions in peak heart rate and peak power output in hypoxic versus normoxic GXT (*p* < 0.05). The NW group showed a greater decline in peak oxygen uptake $\left(\dot{V}O_{2}\text{peak}\right)$ under hypoxia compared to OW (Δ = 9.88 ± 5.0 vs. 5.22 ± 3.3 mL · kg · min^−1^; *p* < 0.001). OW exhibited increased blood glucose levels post‐hypoxic GXT compared to normoxic conditions (Δ = 0.358 mmol · L^−1^; *p* = 0.025). During HIIE sessions, both groups showed similar heart rate, oxygen consumption, carbon dioxide production and respiratory exchange ratio responses. However, blood lactate concentration immediately after normoxic HIIE (NR) was higher in NW compared to OW (*p* < 0.05). Fasting blood glucose significantly increased immediately after normoxic HIIE in NW and immediately after hypoxic HIIE in OW (*p* < 0.05). HIIE in normoxia and hypoxia elicits similar physiological responses across BMI categories, though normal‐weight individuals have greater reductions in $\dot{V}O_{2}\text{peak}$ and higher lactate responses during normoxic HIIE (NR), whereas overweight/obese individuals exhibit higher glucose increases post‐hypoxic exercise, indicating potential BMI‐specific metabolic benefits. These findings suggest that BMI could influence physiological adaptations in response to high‐intensity exercise in hypoxia, suggesting that this form of exercise could be a beneficial alternative for improving metabolic health, especially in individuals with overweight or obesity.

## Summary

- Individuals with a normal BMI show greater $\dot{V}O_{2}\text{peak}$ reductions during hypoxic graded exercise testing (GXT) and elevated lactate responses during normoxic high‐intensity interval exercise (HIIE), highlighting BMI‐dependent responses to exercise.
- Exercise performed under moderate hypoxia elicits more pronounced physiological responses than intensity‐matched exercise in normoxic conditions, suggesting that hypoxic exposure amplifies exercise‐induced stress.
- The heightened glucose and lactate responses observed under hypoxia reflect increased metabolic demand, which may confer health benefits by enhancing glucose regulation and promoting overall metabolic health.

## 1 Introduction

The global obesity epidemic poses a significant public health challenge. Body mass index (BMI), calculated from height and weight, strongly correlates with body fatness (Caterson and Gill 2002). Individuals with a BMI above 25 kg · m^−2^ are classified as overweight, whereas those above 30 kg · m^−2^ are considered obese (Caterson and Gill 2002). Excess body fat increases the risk of diseases, including type 2 diabetes, hypertension and cardiovascular conditions (Atakan, Koşar, et al. 2021).

Aerobic exercise is widely recognised as an effective strategy for combating obesity (Atakan, Koşar, et al. 2021). Among various exercise forms, moderate‐intensity continuous training (MICT) is well‐studied in improving maximal fat oxidation and overall health (Yin et al. 2023). However, significant weight loss with MICT typically requires prolonged exercise durations, with recommendations of at least 150 min per day or 1000 min per week, which can be challenging for those with time constraints (Tate et al. 2007). High‐intensity interval exercise (HIIE) has emerged as a time‐efficient alternative to MICT and involves alternating bursts of intense activity performed at an intensity corresponding to a value ≥ 75% of maximal oxygen uptake $\left(\dot{V}O_{2}\;\max\right)$, with brief rest or lower‐intensity recovery periods (Atakan, Li, et al. 2021). Growing evidence suggests that HIIE could lead to comparable or even superior improvements in cardiorespiratory fitness and metabolic health compared to MICT, with relatively less time commitment (Atakan, Li, et al. 2021; Atakan et al. 2022; Wewege et al. 2017).

Hypoxia, characterised by reduced oxygen availability, places unique stress on the body, triggering adaptive responses to restore homoeostasis (Kayser and Verges 2013). This lower oxygen availability drives specific muscular adaptations, such as increased oxidative enzyme activity (e.g., citrate synthase), mitochondrial density and fibre cross‐sectional area (Hoppeler et al. 2008). Although exposure to severe hypoxia (oxygen level < 10%) may cause adverse health effects, including cognitive impairment and respiratory depression (Allwood et al. 2018), moderate hypoxia (fraction of inspired oxygen [F_i_O_2_] = 12%–16%) was recognised as a therapeutic strategy when combined with exercise. Furthermore, hypoxic exercise can induce further health benefits for individuals with obesity, such as increased energy expenditure, improved insulin sensitivity and favourable changes in body composition, even at lower absolute exercise loads compared to normoxic exercise (Netzer et al. 2008; Hobbins et al. 2021; Kong et al. 2014). In addition, HIIE performed under hypoxic conditions has attracted increasing attention as an effective intervention, due to its ability to elicit significant metabolic demands even at lower relative intensities. Research has shown that high‐intensity exercise performed under hypoxic conditions induced greater energy expenditure and fat oxidation compared to both normoxic exercise and moderate‐intensity exercise in hypoxia (Hoppeler et al. 2008; Millet et al. 2016). These benefits may be especially advantageous for individuals with overweight or obesity, who often struggle to sustain the high mechanical load required for traditional HIIE protocols (Millet et al. 2016). Moreover, performing hypoxic HIIE using nonweight bearing modalities, such as stationary cycling, can reduce joint stress, thereby lowering the risk of musculoskeletal injuries compared to weight‐bearing activities such as running or walking (Netzer et al. 2008). Despite its potential benefits, there is a lack of comprehensive research on the physiological effects of acute HIIE in hypoxia, particularly for individuals with overweight or obesity.

The physiological response to exercise is influenced by various modifiable factors, including exercise intensity, duration, frequency, mode and environmental conditions (Ross et al. 2019). Moreover, other factors, such as age, gender, genetics, BMI and fitness level are known to contribute to inter‐individual variability in exercise responses (Viecelli and Ewald 2022). However, it remains unclear whether BMI (i.e., the distinction between normal‐weight individuals and those with overweight/obesity) significantly affects physiological responses to acute HIIE, either independently or in combination with hypoxia. Therefore, this study employed a moderate hypoxic oxygen level (FiO_2_ = 14%), consistent with a previous study (Y. Li et al. 2022), to investigate the acute physiological responses to HIIE under both normoxic and hypoxic conditions. Furthermore, we examined whether BMI status modulates these responses. We hypothesised that hypoxic HIIE (HY) would induce greater acute physiological responses (e.g., higher exercise heart rate [HR], rating of perceived exertion [RPE] and blood lactate concentration) compared to normoxic HIIE matched for absolute intensity (NA), whereas eliciting similar responses to normoxic HIIE matched for relative intensity (NR). In addition, individuals with overweight and obesity would demonstrate greater physiological strain during hypoxic HIIE compared to normal‐weight individuals.

## 2 Methods

### 2.1 Participants

Twenty‐one healthy young males were recruited from various sources, including social media, nearby universities and the surrounding community. They were divided into two groups: normal‐weight (NW) (*n* = 9, BMI = 22.9 ± 2.3 kg · m^−2^, age = 28.4 ± 3.1 years and peak oxygen uptake $\left[\dot{V}O_{2}\text{peak}\right]$ = 45.1 ± 8.8 mL · kg^−1^ min^−1^) and overweight to moderate obesity (OW) (*n* = 12, BMI = 27.6 ± 2.0 kg · m^−2^, age = 32.3 ± 7.0 years and $\dot{V}O_{2}\text{peak}$ = 39.7 ± 7.7 mL · kg^−1^ min^−1^). Some data from the NW group have been published previously (Y. Li et al. 2022). This sample size is consistent with prior studies investigating the effects of exercise in hypoxia on physiological variables across diverse populations (Hobbins et al. 2021). All participants were nonsmokers with no history of metabolic diseases or hypoxic exposure in the 3 months preceding the study.

### 2.2 Research Design

**FIGURE 1:**
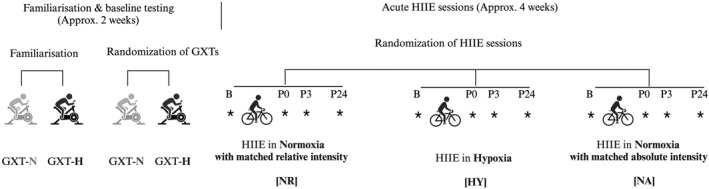
Study design. B, baseline; GXT, graded exercise test; GXT‐H, GXT in hypoxia; GXT‐N, GXT in normoxia; HIIE, high‐intensity interval exercise; HY, hypoxia; NA, normoxia matched for absolute intensity with hypoxia; NR, normoxia matched for relative intensity with hypoxia; P0, immediately post‐exercise; P24, 24 h post‐exercise; P3, 3 h post‐exercise. *Venous blood collection.

The study design, illustrated in Figure 1, involves 8–10 visits to the research laboratory over a 6‐week period as previously described (Y. Li et al. 2022). During the initial visit, participants were familiarised with the testing procedures, followed by four graded exercise tests (GXTs): two in normoxia (F_i_O_2_ = 20.9%, equivalent to standard oxygen levels at sea level) and two in hypoxia (F_i_O_2_ = 14.0%, simulating 3400 m altitude). Following a 1‐week break, participants performed three HIIE sessions (NA, HY and NR) in a randomised order, spaced 1‐week apart. These sessions were scheduled in the morning between 8:00 and 10:00 AM. HR and expired gas were continuously recorded throughout the GXTs and HIIE sessions. Venous blood samples were collected before, immediately after, 3 h and 24 h after each HIIE session and analysed for blood lactate and glucose concentrations (Figure 1). Area under the curve (AUC) for lactate and glucose concentrations was calculated using the trapezoidal method. Participants were instructed to avoid caffeine, alcohol and exercise 24 h before the GXTs and 48 h before the HIIE sessions. Energy requirements for each participant were calculated using the Mifflin–St Jeor equation, based on individual body mass, height and age (Mifflin et al. 1990). Participants received a standardised diet consisting of approximately 53%–56% of total energy from carbohydrates, 22%–24% from fats and 18%–21% from protein (Y. Li et al. 2022).

### 2.3 Familiarisation Procedures and Graded Exercise Test

All participants underwent familiarisation sessions of GXT in both normoxia and hypoxia, with at least 24 h between the sessions. Following this, participants completed four GXTs on an electronically braked cycle ergometer (Excalibur, v2.0; Lode, Groningen, The Netherlands)—two in hypoxia and two in normoxia—in a randomised order, with a minimum of 48 h between each GXT. If peak power output (PPO) differed by more than 10% under the same conditions (e.g., normoxia or hypoxia), an additional GXT was conducted. GXT protocol consisted of 4 min stages at a constant power output, with 30 s rest intervals. To ensure all participants completed eight to nine consecutive 4 min stages in normoxia, the GXTs commenced at 30–60 W, with 15–30 W increments every 4 min based on participants' fitness level (Y. Li et al. 2022; Yan et al. 2018). Capillary blood samples were collected from the fingertip before the test and during rest periods to measure blood lactate concentration using a lactate analyser (YSI 2500 Stat, Yellow Springs, USA). HR was continuously monitored using a HR monitor (Polar H10, Polar Electro Oy, Kempele, Finland), with the highest recorded HR identified as peak HR (HR_peak_). The test ended when participants reached a RPE of 20 on the Borg scale, their cadence dropped below 60 rpm or they voluntarily stopped cycling. The lactate threshold (LT, expressed in *W*) was determined using the modified D‐max formula from the lactate data (Bishop et al. 1998). PPO was defined as the highest power output achieved in the last completed stage. If the last stage was incomplete, PPO was estimated with a modified version of the equation developed by Kuipers et al., that is, PPO = *W*_com_ + (*t*/240 × *W*_inc_), where *W*_com_ represents the *W* of the last completed stage; *t* denotes the time (in seconds) that the final uncompleted stage was sustained and *W*_inc_ is the workload increment at each stage of GXT.

### 2.4 High‐Intensity Interval Exercise Sessions

All participants completed three HIIE sessions (NA, HY and NR) in a randomised order on an electronically braked cycle ergometer (Velotron, RacerMate, Seattle, WA, USA). Each HIIE session commenced with a 5 min warm‐up at 50 W, followed by six 4 min cycling intervals, each separated by 2 min of passive recovery. Immediately after each internal, participants rated their RPE using the Borg scale (6–20) (Borg 1982). Intensity for the 4 min cycling intervals was determined using the formula (LT + 50% × [PPO − LT]) (Bishop et al. 1998). For the HY session, the intensity was based on PPO (PPO_H_) and lactate threshold (LT_H_) from the GXTs performed in hypoxia (50% PPO_H_ + 50% LT_H_). For the NR session, the intensity was based on PPO (PPO_N_) and the LT_N_ achieved in normoxia (50% PPO_N_ + 50% LT_N_). For the NA session, the exercise intensity matched HY, equating to 50% PPO_H_ + 50% LT_H_. During the HY session, participants spent approximately 80 min inside the hypoxia chamber, which included a 30 min acclimation phase, a 5 min warm‐up at 50 W, a 34 min HIIE session and a 5 min post‐exercise recovery.

### 2.5 Cardio‐Respiratory Responses

During both the GXT and HIIE sessions, oxygen consumption $\left(\dot{V}O_{2}\right)$ and carbon dioxide production $\left(\dot{V}{\text{CO}}_{2}\right)$ were recorded using a gas analyser (Moxus 2010, AEI Technologies Inc., Pittsburgh, PA, USA). Prior to each test, the gas analyser was calibrated using a 3L syringe (Hans Rudolph, Shawnee, KS, USA) and gas verification was conducted using known concentrations of O_2_ (16.2% and 20.9%, respectively) and CO_2_ (0% and 4.0%, respectively). Measurements of $\dot{V}O_{2}$, $\dot{V}{\text{CO}}_{2}$ and respiratory exchange ratio (RER) were recorded at 15 s intervals. The highest values of oxygen consumption and carbon dioxide production recorded within any 60 s interval during the GXT were identified as the participant's peak $\dot{V}O_{2}\left(\dot{V}O_{2}\text{peak}\right)$ and peak $\dot{V}{\text{CO}}_{2}\left(\dot{V}{\text{CO}}_{2}\text{peak}\right)$, during HIIE sessions, these values were represented by $\dot{V}O_{2}$ and $\dot{V}{\text{CO}}_{2}$, respectively.

### 2.6 Statistical Analysis

Data distribution was evaluated using the Shapiro–Wilk test. Two‐way repeated‐measures analysis of variance (ANOVA) was conducted to examine the main effects of group (NW or OW), condition (normoxia or hypoxia) and their interaction. Three‐way ANOVA with repeated measures was used to determine the effect of condition (NA, NR or HY), group (NW or OW) and time point (pre to 24 h post‐exercise) on concentrations of glucose and lactate following the HIIE sessions. Where appropriate, post hoc comparisons for three‐way ANOVA were made using simple main effect tests. When Mauchly's test indicated a violation of the sphericity assumption, the Greenhouse–Geisser correction was applied. A potential relationship between concentrations of glucose and lactate was determined with Pearson's product‐moment correlation coefficient. A partial eta‐squared (*η*_*p*_^2^) was reported as a measure of effect size and classified as a small effect (0.01 ≤ *η*_*p*_^2^ < 0.06), a medium effect (0.06 ≤ *η*_*p*_^2^ < 0.14) and a large effect (*η*_*p*_^2^ ≥ 0.14) (Cohen 2013). Data are presented as mean ± standard deviation (SD) as well as mean difference (Δ) and 95% confidence interval (95% CI). Statistical analyses were performed using SPSS version 26.0 (IBM Corp., Armonk, NY, USA), and graphs were made using GraphPad Prism (V8.0, GraphPad Software Inc., San Diego, CA, USA), with significance set at *p* < 0.05.

## 3 Results

The characteristics of participants are presented in Table 1. No significant differences were observed between the two groups in age, height, $\dot{V}O_{2}\text{peak}$, PPO and HR_peak_ from GXTs under normoxic conditions. As expected, the OW group had significantly higher body weight and BMI than the NW group (*p* < 0.01) (Table 1).

**TABLE 1:** Participants' characteristics.

Variables	NW (*n* = 9)	OW (*n* = 12)	*t*	*p*
Age (years)	28.4 ± 3.1	32.3 ± 7.0	1.684	0.112
Body weight (kg)	69.8 ± 8.3	92.7 ± 13.3	−4.547	< 0.001
Body height (m)	1.75 ± 0.07	1.83 ± 0.12	−1.908	0.054
BMI (kg · m^−2^)	22.9 ± 2.3	27.6 ± 2.0	−4.970	< 0.001
$\dot{V}O_{2}\text{peak}$ (L · min^−1^)	3.19 ± 0.91	3.65 ± 0.79	−1.255	0.237
$\dot{V}O_{2}\text{peak}$ (mL · kg^−1^ min^−1^)	45.1 ± 8.8	39.7 ± 7.7	1.521	0.155
Peak power output (Ẇ_p_)	212 ± 70	272 ± 67	−1.972	0.066
Peak power output/body mass (Ẇ_p_ · kg^−1^)	3.00 ± 0.68	2.94 ± 0.67	0.178	0.861
HR_peak_ (beats · min^−1^)	187 ± 7	180 ± 10	1.867	0.063

*Note:* Values are mean ± standard deviation.

Abbreviations: BMI, body mass index; HR, heart rate; NW, normal weight; OW, overweight/obese; $\dot{V}O_{2}\text{peak}$, peak oxygen uptake; Ẇ, watt.

### 3.1 Comparison of Physiological Responses to GXTs in Normoxia and Hypoxia Between Individuals With Normal‐Weight and Overweight/Obesity

**FIGURE 2:**
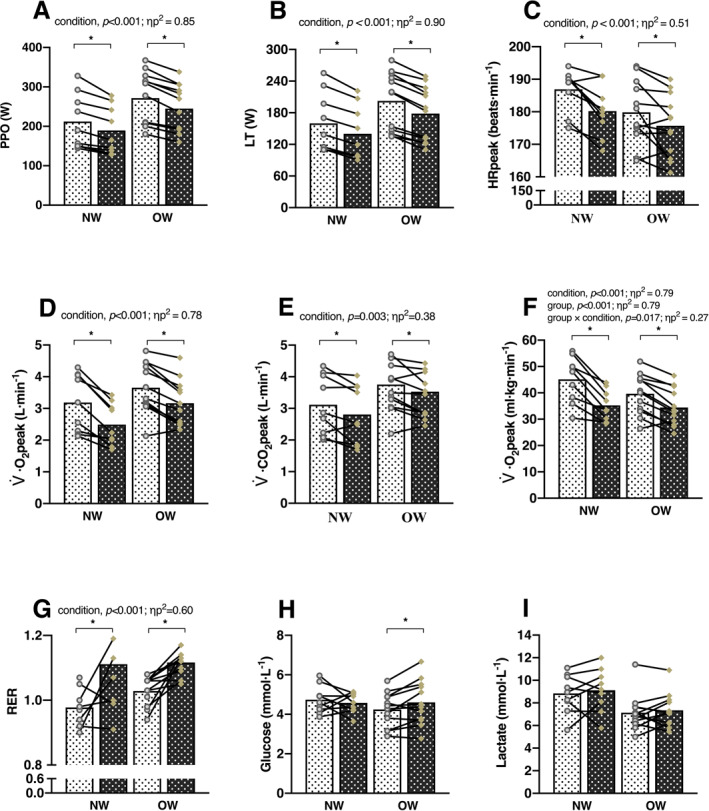
Physiological responses to GXTs in normoxia and hypoxia among adults with normal‐weight (NW) and those with overweight or obesity (OW). Symbols connected by lines represent individual participants, with solid grey circles denoting the normoxia condition and coloured diamonds denoting the hypoxia condition. *p < 0.05. *η*_*p*_^2^, partial eta squared; (A) PPO, peak power output; (B) LT, lactate threshold; (C) HR_peak_, peak heart rate; (E) $\dot{V}{\text{CO}}_{2}\text{peak}$, volume of peak carbon dioxide production; (D, F) $\dot{V}O_{2}\text{peak}$, peak oxygen uptake. (G) RER, respiratory exchange ratio. (H) Blood glucose level. (I) Blood lacate level.

Data from hypoxic GXTs showed significantly lower values for PPO, LT, HR_peak_, $\dot{V}{\text{CO}}_{2}\text{peak}$ and $\dot{V}O_{2}\text{peak}$ in both groups compared to normoxic GXTs (*p* < 0.05) (Figure 2). There were no significant differences between groups or any group × condition interactions for these variables. A significant group × condition interaction was observed for $\dot{V}O_{2}\text{peak}$ (*p* = 0.017; *η*_*p*_^2^ = 0.27) (Figure 2F), with a greater reduction in $\dot{V}O_{2}\text{peak}$ from normoxia to hypoxia in the NW group (NW: 20.35%, Δ = 9.88 ± 5.0 mL · kg · min^−1^ vs. OW: 12.82%, Δ = 5.22 ± 3.3 mL · kg · min^−1^, *p* < 0.001; *η*_*p*_^2^ = 0.791). Both NW (Δ = 0.13; [95% CI 0.04 to 0.23] and *p* = 0.012) and OW groups (Δ = 0.09; [95% CI 0.05 to 0.13] and *p* < 0.001) had a higher RER in hypoxia compared to normoxia, with no significant group difference between groups or group × condition interaction (Figure 2G). Peak blood glucose concentration increased significantly only in the OW group after the GXT in hypoxia compared to normoxia (Δ = 0.36 mmol · L^−1^; [95% CI 0.05 to 0.66]; *p* = 0.025), whereas no significant change was observed in the NW group (Figure 2H). Additionally, blood lactate concentration did not differ significantly between conditions in either group and no group × condition interactions were observed (Figure 2I).

### 3.2 Comparison of Physiological Responses to HIIE in Normoxia and Hypoxia Between Individuals With Normal‐Weight and Overweight/Obesity

The intensities of HIIE sessions in the NW group were 186 ± 62 W and 165 ± 56 W for NR and HY/NA conditions, respectively (*p* < 0.001) (Table 2). In contrast, the OW group had higher intensities of 236 ± 60 W and 212 ± 57 W for NR and HY/NA, respectively (*p* < 0.001) (Table 2). The power output relative to body weight was higher in NR conditions when compared to NA/HY conditions for both NW and OW groups (*p* < 0.001). RPE was significantly higher after HIIE sessions in HY and NR compared to HIIE in NA (*p* < 0.05), with no differences between HY and NR (*p* > 0.05). No main effects of group or group × condition interactions were observed (Table 2). In the NW group, HR_peak_ was significantly higher after HIIE sessions in normoxia and hypoxia compared to HIIE in NA (*p* < 0.05) (Table 2). In the OW group, HR_peak_ was significantly higher following HIIE in NR compared to NA (*p* = 0.011) (Table 2), with no significant differences in HR_peak_ across the other conditions (*p* > 0.05). In the NW group, $\dot{V}O_{2}$ was significantly higher after HIIE in NR compared to HIIE in NA (*p* = 0.043) (Table 2), with no significant differences observed in the other comparisons (*p* > 0.05) (Table 2). In the OW group, $\dot{V}O_{2}$ did not differ significantly across conditions (*p* > 0.05) and no significant differences in $\dot{V}{\text{CO}}_{2}$ were observed across conditions in either group (*p* > 0.05) (Table 2). No significant differences in RER were observed across conditions in the NW group (Table 2). However, in the OW group, RER was significantly higher during HIIE in HY compared to HIIE in NA (*p* = 0.020) and HIIE in NR (*p* = 0.048) (Table 2).

**TABLE 2:** Physiological responses to HIIE in normoxia and hypoxia in individuals with normal BMI and overweight/obesity.

Variables	NW (*n* = 9)			OW (*n* = 12)			Group		Condition		Group × condition	
	NA	HY	NR	NA	HY	NR	*p*	*η*_*p*_^2^	*p*	*η*_*p*_^2^	*p*	*η*_*p*_^2^
50%LT + 50%PPO (W)	165 ± 56*	165 ± 56*	186 ± 63	212 ± 57*	212 ± 57*	236 ± 60	0.075	0.157	< 0.001	0.888	0.396	0.038
50%LT + 50%PPO/weight (Ẇ · kg^−1^)	1.97 ± 0.52*	1.97 ± 0.52*	2.25 ± 0.57	1.93 ± 0.52*	1.93 ± 0.52*	2.20 ± 0.57	0.862	0.002	< 0.001	0.907	0.840	0.009
RPE	16 ± 2.3	18 ± 2.5^#^	19 ± 2.2^#^	16 ± 2.1	18 ± 1.9^#^	18 ± 2.6^#^	0.968	0.000	< 0.001	0.556	0.230	0.074
HRpeak (beats · min^−1^)	161 ± 14	172 ± 10^#^	178 ± 9^#^	161 ± 13	168 ± 11	169 ± 9^#^	0.343	0.047	< 0.001	0.508	0.123	0.104
$\dot{V}O_{2}$ (mL · kg · min^−1^)	35.5 ± 8.0	32.8 ± 7.0	43.3 ± 7.0^#^	33.5 ± 7.6	31.4 ± 9.1	35.1 ± 8.5	0.278	0.078	0.003	0.318	0.161	0.114
$\dot{V}{\text{CO}}_{2}$ (mL · kg · min^−1^)	34.0 ± 8.3	34.9 ± 7.5	41.3 ± 7.3	30.1 ± 5.8	30.8 ± 6.6	32.7 ± 7.4	0.077	0.194	0.020	0.230	0.332	0.071
RER	0.96 ± 0.03	1.07 ± 0.11	0.95 ± 0.08	0.91 ± 0.08	1.00 ± 0.08* ^,^ ^#^	0.94 ± 0.06	0.132	0.145	< 0.001	0.436	0.572	0.037

Abbreviations: *η*_*p*_^2^, partial eta squared; HRpeak, peak heart rate; HY, hypoxia; NA, normoxia matched for absolute intensity with hypoxia; NR, normoxia matched for relative intensity with hypoxia; RER, respiratory exchange ratio; RPE, ratings of perceived exertion; $\dot{V}{\text{CO}}_{2}$, volume of peak carbon dioxide production; $\dot{V}O_{2}$, volume of peak oxygen uptake.

*Significant difference versus NR within the same group (*p* < 0.05).

^#^Significant difference versus NA within the same group (*p* < 0.05).

**FIGURE 3:**
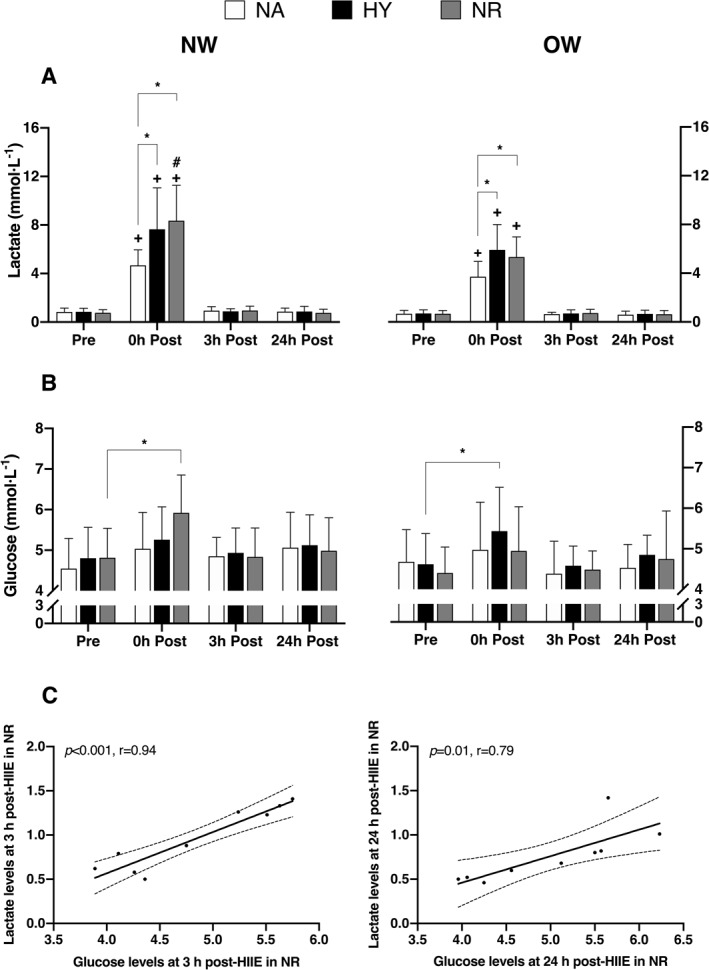
Blood lactate (A) and glucose concentrations (B) and their relationship (C) following high‐intensity interval exercise under normoxic and hypoxic conditions among adults with normal‐weight (NW) and those with overweight or obesity (OW). Values are presented as means ± SD. 0 h Post, immediately after exercise; 3 h Post, 3 h post‐exercise; 24 h Post, 24 h post‐exercise; HY, hypoxia; NA, normoxia matched for absolute intensity with hypoxia; NR, normoxia matched for relative intensity with hypoxia; Pre, baseline. *p < 0.05. +represents a significant difference compared to pre within the same condition. #shows the significant difference compared to the OW group at the same time point within the same condition.

**FIGURE 4:**
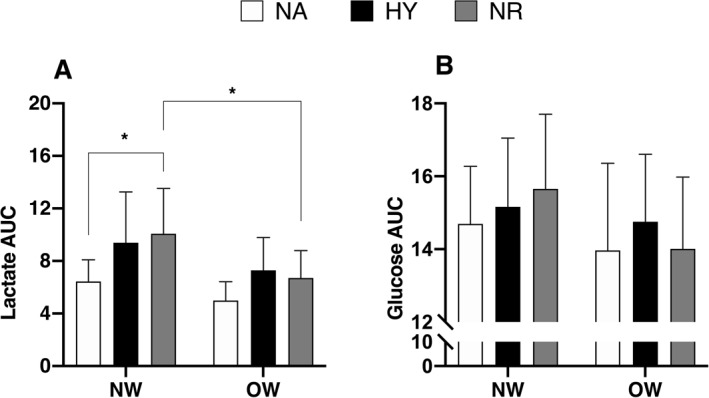
Area under the curve (AUC) values for blood lactate (A) and glucose concentrations (B) following HIIE under normoxic and hypoxic conditions among adults with normal‐weight (NW) and those with overweight or obesity (OW). Values are presented as means ± SD. HY, hypoxia; NA, normoxia matched for absolute intensity with hypoxia; NR, normoxia matched for relative intensity with hypoxia. *p < 0.05.

Three‐way ANOVA with repeated measures revealed a significant main effect of HIIE (*p* < 0.001) as well as a significant main effect of time point (*p* < 0.001) on blood lactate concentrations, indicating increased levels post‐HIIE (Figure 3A). Further analysis of the time point × condition interaction showed significantly greater lactate concentrations following HIIE in both hypoxia and normoxia with relative‐matched intensity compared to HIIE in NA (*p* < 0.001; Figure 3A). Normal‐weight participants exhibited significantly higher post‐HIIE lactate concentrations in NR (8.4 ± 2.9 mmol · L^−1^) compared to those with overweight or obesity (5.3 ± 1.6 mmol · L^−1^; *p* < 0.001). No significant between‐group differences were observed in hypoxia or NA conditions (Figure 3A). For blood glucose concentrations, there was a significant main effect of time point (*p* < 0.001; Figure 3B). Post hoc analysis revealed condition‐specific responses: in the NW group, glucose levels increased significantly immediately after HIIE in normoxia compared to baseline (Δ = 1.11 mmol · L^−1^; *p* = 0.011). In the OW group, glucose concentrations increased significantly after HIIE in hypoxia compared to baseline (Δ = 0.815 mmol · L^−1^; *p* = 0.017) (Figure 3B). Lactate and glucose concentrations were significantly correlated at 3 h (*r* = 0.94 and *p* < 0.001) and 24 h (*r* = 0.79 and *p* < 0.05) after HIIE in normoxia for the NW group (Figure 3C). The AUC for blood lactate was significantly higher in the NW group following HIIT in normoxia compared to both HIIE in NA and HIIE in normoxia in the OW group (*p* < 0.05; Figure 4A). There was no significant difference in the AUC for blood glucose among the trials in either group (Figure 4B).

## 4 Discussion

In this study, we investigated the impact of GXT and HIIE in hypoxia on physiological and perceptual responses in individuals with normal‐weight and overweight/obesity. Our results show that performing the GXT in hypoxia resulted in a more pronounced reduction in $\dot{V}O_{2}\text{peak}$ in normal‐weight individuals compared to those with overweight/obesity. In addition, HIIE in hypoxia elicited greater changes in RPE and blood lactate concentrations than normoxic HIIE matched for absolute intensity in both NW and OW groups. The NW group exhibited higher lactate responses than the OW group immediately after HIIE under the NR condition, which may suggest that the lactate response to higher‐intensity exercise is influenced by BMI status. However, when normoxic HIIE was matched for relative intensity of hypoxia (approximately 12% higher power output than HY), physiological responses (RPE, HR, glucose and lactate concentrations) were similar across both groups. These findings suggest that incorporating hypoxic stress into HIIE may induce more pronounced physiological changes, and that exercising at lower absolute intensities in hypoxia could elicit comparable physiological responses to higher intensities in normoxia, largely independent of BMI. Importantly, these effects being independent of BMI would suggest that hypoxic exercise could be effectively integrated into weight management programmes, offering metabolic and cardiovascular benefits without the need for strenuous workouts.

Both NW and OB groups experienced a significant decrease in $\dot{V}O_{2}\text{peak}$ during GXTs, consistent with previous research showing reduced $\dot{V}O_{2}\text{peak}$ and maximal power during GXT in normobaric hypoxia (F_i_O_2_ = 0.125, equivalent to 4000 m simulated altitude) in untrained individuals (Gallagher et al. 2014). The reduction in $\dot{V}O_{2}\text{peak}$ during GXTs in hypoxia was more pronounced in lean individuals than in those with overweight or obesity, when compared to GXTs performed in normoxia. Although $\dot{V}O_{2}\text{peak}$ in NW was 13.8% higher than that of OW ($\dot{V}O_{2}\text{peak}$ = 45.1 ± 8.8 mL · kg · min^−1^ vs. 39.7 ± 7.7 mL · kg · min^−1^), the difference was not statistically significant. $\dot{V}O_{2}\;\max/\dot{V}O_{2}\text{peak}$ is widely recognised as a key indicator of aerobic capacity and cardiovascular fitness, influenced by factors such as pulmonary system, maximum cardiac output, oxygen‐carrying capacity and skeletal muscle function (Bassett and Howley 2000). The greater reduction in $\dot{V}O_{2}\text{peak}$ observed in normal‐weight individuals may be attributed to factors such as reduced arterial O_2_ content from desaturation, impaired pulmonary gas exchange and decreased maximal cardiac output and peak leg blood flow (Calbet et al. 2003). Notably, individuals with higher physical activity levels typically exhibit higher $\dot{V}O_{2}\;\max$ values than sedentary individuals, reflecting differences in fitness levels and oxygen transport efficiency (Wagner 2000). The adverse effects of hypoxia on aerobic performance tend to be more pronounced in individuals with higher aerobic capacity due to greater hypoxaemia (Kayser and Verges 2013; Chapman et al. 1999). Further research is needed to elucidate the mechanisms behind the varying reductions in $\dot{V}O_{2}\text{peak}$ among individuals with different BMIs.

Another key finding was that both lean adults and individuals with overweight or obesity exhibited similar increases in RPE and blood lactate concentration immediately after HIIE sessions in hypoxia and in normoxia matched for relative intensity, with greater changes than in normoxia matched for absolute intensity. This suggests that combining hypoxia with HIIE could amplify metabolic stress, even at lower exercise intensities, as both groups experienced comparable metabolic stress. These findings align with previous research reporting no difference in RPE between maximal power output in normoxia and 85% of maximal normoxic output in hypoxia (Chacaroun et al. 2019). However, in endurance athletes ($\dot{V}O_{2}\;\max$: 62.5 ± 1.2 mL · kg · min^−1^), Sumi et al. (Sumi et al. 2019) reported a greater increase in blood lactate levels immediately after HIIE (10 × 3 min run at 90% of $\dot{V}O_{2}\;\max$ with 1 min active rest) under similar hypoxia condition (F_i_O_2_ = 14.5%) with the same relative intensity compared to normoxia. This discrepancy may be attributed to the varying fitness levels of the participants, as individuals with higher aerobic capacity tend to experience greater metabolic perturbations under hypoxic conditions. The absence of significant between‐group differences in lactate response under hypoxic conditions may be attributable to participants' untrained status and relatively low baseline aerobic capacity. However, in normoxic conditions, the NW group exhibited significantly higher lactate concentration compared to OW group under NR, consistent with previous findings (Rodriguez et al. 2018). This between‐group difference was not observed in NA, suggesting that higher exercise intensity may preferentially enhance glycolytic contribution to energy metabolism in NW individuals instead of the OW group. In addition, the strong correlation between blood and glucose concentrations that only observed in 3 h post and 24 h post‐HIIE under NR in the NW group. This may reflect the interdependent regulation of glucose and lactate metabolism during recovery phase, given that lactate, a byproduct of glycolysis, is produced when hypoxia limits ATP generation via the tricarboxylic acid cycle, prompting a shift toward anaerobic metabolism (X. Li et al. 2022). Following HIIE, lactate accumulation is cleared primarily through oxidation in skeletal muscle and other tissues. Additionally, the Cori cycle facilitates the conversion of lactate back to glucose in the liver, contributing to the maintenance of glucose homoeostasis (Bartoloni et al. 2024). The strong correlation may indicate that individuals with normal‐weight exhibit greater metabolic flexibility compared to those with overweight or obesity, allowing for more efficient and coordinated regulation of glucose and lactate in response to HIIE under normoxic conditions. We observed a significant increase in blood glucose concentration in NW after HIIE matched for relative intensity (i.e., NR condition) compared to HIIE matched for absolute intensity (i.e., NA condition). This immediate response suggests a greater impact on glycogen depletion and glucose mobilisation at higher exercise intensities in the NW group. Notably, the blood glucose response to HIIE in NA was comparable between individuals with normal‐weight and those with overweight and obesity, suggesting a consistent glucose response regardless of body weight. However, individuals with overweight or obesity experienced a significant increase in blood glucose levels after HIIE in hypoxia, whereas no such increase was observed in NW participants. Typically, exercising in hypoxia at the same absolute intensity as normoxia results in a higher relative intensity, leading to increased muscle glycogen utilisation and depletion (Girard et al. 2017). Additionally, evidence suggests that hypoxia alone can stimulate glucose uptake in a manner similar to exercise‐induced muscle contractions (Calbet et al. 2003; Soo et al. 2023). This implies that hypoxia may activate key signalling pathways responsible for glucose transport, enhancing the exercise stimulus and promoting improved glucose regulation. The difference in blood glucose responses between NW and OW groups suggest that the underlying regulatory mechanisms may differ. The elevated blood glucose levels in the OW group could be attributed to higher adrenaline levels, which facilitate glycogenolysis in both the liver and skeletal muscle following high‐intensity exercise (Kindermann et al. 1982). Our findings may indicate that exercising in hypoxia at the same relative intensity as normoxia (i.e., lower absolute intensity) induces comparable effects on glucose metabolism in individuals with overweight or obesity, a pattern that may not extend to those with normal weight. Overall, hypoxic exercise may serve as an effective strategy to improve muscle glycogen utilisation and glucose uptake in individuals with overweight or obesity, particularly given the increased relative intensity of exercise in hypoxia, which may enhance glucose regulation (Soo et al. 2023) without requiring an excessive workload. Furthermore, hypoxia may synergistically enhance glucose transport activity, similar to the effects of high‐intensity exercise (van der Zwaard et al. 2018). This could provide a safe and effective alternative for improving glucose metabolism in this population. Nevertheless, further research is needed to fully understand the mechanisms underlying the effects of high‐intensity interval exercise in hypoxia on glucose metabolism in individuals with overweight or obesity.

The present study has several methodological limitations. First, although participants were instructed to maintain their regular physical activity levels, future research should incorporate accelerometer to monitor incidental activity. Furthermore, the timing of exercise tests can influence performance outcomes (Ayala et al. 2021). Although participants were instructed to perform tests at the same time of day, adherence was inconsistent, necessitating repeated GXTs until the PPO difference was less than 10% to ensure reliability. Another limitation is the exclusion of female participants, which restricts the generalisability of our findings to females. Future studies should include both sexes to determine whether similar responses occur in female participants. Additionally, our study focused on healthy individuals, limiting its applicability to clinical populations. Future research should recruit individuals with medical conditions, such as diabetes, metabolic syndrome or chronic obstructive pulmonary disease (COPD), to gain broader understanding of the physiological response to HIIE in hypoxia. Finally, our study focused on HIIE in moderate hypoxia, necessitating future research on the effects of different hypoxia severities and/or different patterns of hypoxic exposure (i.e., inter‐effort recovery hypoxia) (Papoti et al. 2023).

## 5 Conclusion

This study demonstrates that moderate hypoxia can enhance the acute physiological and perceptual responses to high‐intensity interval exercise, even when performed at absolute workloads. Individuals with a normal BMI experienced a greater reduction in $\dot{V}O_{2}\text{peak}$ during hypoxic GXT and higher lactate responses when HIIE performed in normoxia with workload matched to relative hypoxic intensity, suggesting BMI‐dependent differences in metabolic response during HIIE in normoxia. However, when exercising at a lower absolute exercise intensity in hypoxia, the incorporation of hypoxic stress elicited greater physiological responses than HIIE alone (NA), resulting in similar changes to HIIE with relatively matched intensity of hypoxia regardless of body compositions. This finding supports that HIIE in hypoxia could serve as an effective alternative method for individuals struggle with high‐intensity mechanical loads.

## Author Contributions

J.L., D.J.B., Y.L., X.Y. and L.P. contributed to the study design and conception. Z.W., J.L., M.M.A. and X.Y. participated in data acquisition. Z.W. performed the data analyses. J.L., M.M.A., M.K., H.L., J.K., M.J.M., W.L., O.G., X.Y. and P.L. contributed to the interpretation of the results. Z.W., M.M.A., X.Y. and L.P. wrote the original manuscript. All authors critically reviewed the manuscript, approved its final version for publishing and agreed to be accountable for all aspects of this work.

## Ethics Statement

This study received ethical approval from the Victoria University Human Research Ethics Committee (Approval No: HRE18‐214), and all procedures complied with the principles of the Declaration of Helsinki.

## Conflicts of Interest

The authors declare no conflicts of interest.

## Data Availability

The data will be made available from the author upon reasonable request via email.
